# Application of the international classification of functioning, disability, and health in autism and attention-deficit hyperactivity disorder: A scoping review

**DOI:** 10.1177/13623613241272044

**Published:** 2024-08-25

**Authors:** Lovisa Alehagen, Sven Bölte, Melissa H Black

**Affiliations:** 1 Karolinska Institutet and Region Stockholm, Sweden; 2 Stockholm Health Care Services, Region Stockholm, Sweden; 3 Curtin University, Australia

**Keywords:** attention-deficit hyperactivity disorder, autism, International Classification of Functioning, Disability, and Health, International Classification of Functioning Core Sets, International Classification of Functioning, Disability, and Health-Youth and Child version, scoping review

## Abstract

The International Classification of Functioning, Disability, and Health is a biopsychosocial framework of health-related functioning designed to provide a unifying system for health care, social services, education, and policy sectors. Since its publication in 2001, the International Classification of Functioning has been used to guide clinical practice and research in autism and attention-deficit hyperactivity disorder. More recently, to promote its practical application, shorter versions of the International Classification of Functioning, known as Core Sets, have been developed for autism and attention-deficit hyperactivity disorder. This scoping review explored the application of the International Classification of Functioning and the International Classification of Functioning Core Sets in autism and attention-deficit hyperactivity disorder. Electronic database searches were conducted, resulting in 78 studies meeting the inclusion criteria. The results demonstrate a wide-ranging application of the International Classification of Functioning using different methods across various contexts. However, the majority of the research conducted focused on autism and primarily involved children. The review highlights that although the research implemented the International Classification of Functioning, it did not necessarily align with the underlying philosophy of the International Classification of Functioning framework, as the impact of the medical perspective still seems to influence research methodologies and interpretation. Nonetheless, the International Classification of Functioning presents opportunities to facilitate a shift from a strictly medical viewpoint on neurodevelopmental conditions to more comprehensive approaches.

**Lay abstract:**

The International Classification of Functioning, Disability, and Health (ICF) is a framework designed by the World Health Organization (WHO) to help different sectors, such as healthcare, social services, education, and policy, understand how people with health-related issues function (do the things they want to and need to do) in their daily lives. This framework has also been used to guide clinical practice and research in autism and attention-deficit hyperactivity disorder (ADHD). To make it more practical, shorter versions of the ICF called Core Sets have been developed. We wanted to explore how the ICF and the ICF Core Sets have been used in research relating to autism and ADHD. We looked at the research that had been previously published on this topic by conducting a systematic search and review. Seventy-eight studies meeting our criteria were included in our review. Results show that the ICF has been applied in many ways across various contexts. However, most of the research has focused on autism, mainly involving children. The review highlights that although the ICF was used in some studies, the underlying philosophies of the framework were not always followed. The medical perspective still influenced the way research was done and interpreted. Nevertheless, using the ICF in the right way can help shift research on neurodevelopmental conditions like autism and ADHD toward a more holistic approach, moving away from solely focusing on medical aspects.

Autism and attention-deficit hyperactivity disorder (ADHD) are two common neurodevelopmental conditions (NDCs) (Thomas et al., 2015; Zeidan et al., 2022). Both conditions represent a divergence from typical development and are defined by functioning difficulties that interfere with mainstream social, academic, or occupational demands and expectancies (Levy et al., 2009). Though diagnostic criteria and functioning profiles vary between individuals with autism and ADHD, there is considerable trait overlap (Taurines et al., 2012), the conditions often co-occur (Hollingdale et al., 2020), and many functional challenges are shared (Bölte, de Schipper, Robison, et al. 2014; Bölte et al., 2019). Some estimates have suggested that up to 70% of autistic individuals may have co-occurring ADHD (Hours et al., 2022), while approximately 13% of individuals with ADHD are also diagnosed with autism (Jensen & Steinhausen, 2015; Zablotsky et al., 2020).

The biomedical perspective has largely underpinned research and practice in NDCs, focusing on symptomatology and categorical clinical assessment. This perspective has emphasized individual problems to explain functional difficulties (Provenzani et al., 2020; Selb et al., 2015; Üstün, 2007). Today, however, there is increasing recognition that individual difficulties alone cannot account for challenges experienced by individuals with NDCs (Pellicano & den Houting, 2022). For instance, being considered “high functioning” (no presence of intellectual disability) does not necessarily translate to functional outcomes (Alvares et al., 2020), and the correlation between symptomology and quality of life (QoL) is complex (Oakley et al., 2021; van Heijst & Geurts, 2015), with other factors beyond symptomology found to more strongly predict QoL in autism and ADHD (Coghill & Hodgkins, 2016; Mulraney et al., 2019; Oakley et al., 2021). It is clear that diagnostic status alone does little to capture the lived experience of individuals with NDCs. In recognition of the limitations associated with a sole focus on symptoms and impairment, NDC communities and neurodiversity-aligning researchers are calling for a move away from psychopathology views and terminologies toward more comprehensive perspectives that capture all capabilities of individuals, including strengths and the role of the environment (Pellicano & den Houting, 2022). There is growing acceptance of the neurodiversity paradigm, which argues that NDCs are part of natural human neural diversity, where disability arises from a poor fit between the individual and their environment rather than exclusively from individual difficulties (Walker, 2021). Indeed, research appears to support the notion that environments, such as an individual’s social or physical surroundings, can impact levels of disability or ability (Black et al., 2019; Bölte, Leifler, et al., 2021; Crompton et al., 2020; Lasky et al., 2016).

The World Health Organization’s (WHO) International Classification of Functioning (ICF) aligns with these changing priorities and has received growing attention in NDCs since its publication in 2001 (WHO, 2001). In the WHO classification family, the ICF serves as a complementary framework and classification to the International Classification of Diseases and Related Health Problems (ICD) (WHO, 2022), where the ICD primarily captures diagnosis, and the ICF specifically addresses the concept of functioning. In the ICF, functioning is operationalized as the complex interplay between individuals’ strengths and challenges, and contextual factors that can act as facilitators or barriers (WHO, 2001). A focus on functioning more closely aligns with the research priorities of the NDC community, such as addressing QoL and daily challenges (Dijkhuis et al., 2017). Since the ICF is “diagnostically agnostic,” it considers the environments’ role in functioning and disability and takes into account individual strengths; it aligns directly with neurodiversity perspectives (Bölte, Lawson, et al., 2021; Dwyer, 2022). Neither neurodiversity nor purely biomedical perspectives comprehensively cover all aspects of NDCs, and both approaches have their respective strengths and weaknesses. However, the ICF offers the potential to enhance understanding of NDCs by taking into account biological, psychological, and social factors influencing functioning. It can, therefore, serve as a bridge between these two essential approaches.

The biopsychosocial model of the ICF is designed to provide a shared framework for different professions such as health and social services professionals, educators, and policymakers to understand health-related functioning. The ICF captures body functions (physiological and psychological functions), body structures (anatomical parts of the body), participation and activities (involvement in everyday life and execution of tasks), environmental factors (physical, social, and attitudinal environment), and personal factors (personal background information including gender, age, and social background) (see Figure 1). The ICF classification system is organized using a hierarchical structure across the domains of body functions, body structures, activity and participation, and environmental factors, with codes arranged under four levels with increasing levels of detail. Although personal factor codes are part of the ICF framework and recognized as important in functioning, they are not coded in the ICF, and there is no defined format or structure for their classification. The absence of personal factor codes is grounded in the significant social and cultural variability associated with them (WHO, 2001). The Youth and Child version (ICF-CY), designed to capture aspects of functioning important for developing individuals, represents the most comprehensive version, comprising 1685 codes across the four levels (WHO, 2007).

**Figure 1.**
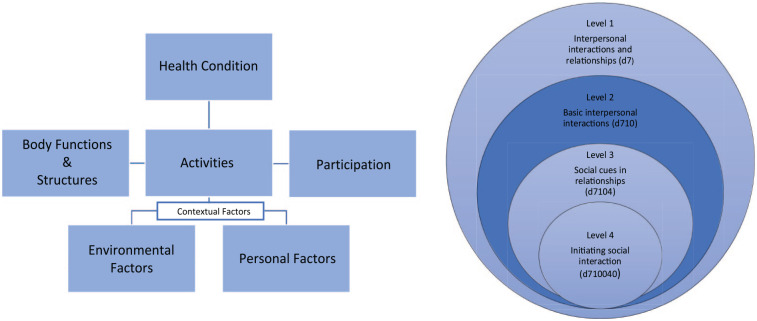
The biopsychosocial model of the ICF (left) and example of the hierarchically organized category structure of the ICF in the activities and participation domain (right). Though the ICF can include up to four levels of detail, the ICF Core Sets are usually restricted to the second level, which is indicated in the darker color.

The ICF has been used internationally to guide policy, assessment, and evaluation of NDCs in several sectors, such as the Australian guidelines for assessment of autism (AutismCRC, 2018), the Federal German Participation Law (Bundesteilhabegesetz, 2016), and is being recommended by the National Health Service (NHS) England for autism and ADHD (Autistica, 2021). Although the ICF has had some recent influence on policy, its impact is still relatively limited. One hindrance is the extensiveness of the framework which makes its use cumbersome in practice. Therefore, shortlists of the ICF, consisting only of the most relevant codes for certain diagnoses or contexts, have been developed, so-called ICF Core Sets. Core Sets for several diagnoses have been published, for example, cerebral palsy (Schiariti et al., 2015), multiple sclerosis (Coenen et al., 2011), depression (Cieza et al., 2004), and schizophrenia (Nuno et al., 2018), as well as autism and ADHD (Bölte et al., 2018, 2019). Typically, Core Set developments follow a rigorous protocol outlined by the WHO that aims to represent various international and stakeholder perspectives (Selb et al., 2015).

The Core Sets for autism and ADHD were published in 2018 and 2019 to guide clinical practice and research. However, it is unknown how the ICF and the autism and ADHD Core Sets have been used in and influenced research and practice to date. For this reason, we conducted a scoping review examining the application of the ICF and ICF Core Sets in autism and ADHD.

## Autism and ADHD Core Sets

The ICF Core Sets for autism and ADHD are based on the ICF-CY and were established according to a rigorous multimethod scientific procedure for ICF Core Set development established by the ICF Research Branch and WHO (Bölte, de Schipper, Holtmann, et al., 2014; Bölte, de Schipper, Robison, et al., 2014; Selb et al., 2015). Four preparatory studies comprising comprehensive systematic literature reviews (de Schipper, Lundequist, Coghill, et al., 2015; de Schipper, Lundequist, Wilteus, et al., 2015), expert surveys (de Schipper, Mahdi, et al., 2015; de Schipper et al., 2016), qualitative studies (Mahdi et al., 2017; Mahdi, Viljoen, et al., 2018), and clinical studies (Mahdi, Albertowski, et al., 2018; Mahdi, Ronzano, et al., 2018) assisted in identifying candidate ICF categories perceived to be important for functioning from relevant stakeholder groups. Consensus conferences for each diagnosis, consisting of a multidisciplinary and international group of experts representing all six WHO regions combined findings of the preparatory studies and developed the final Core Sets for autism and ADHD (Bölte et al., 2018, 2019). These processes yielded 72 ICF codes in the comprehensive Core Set for ADHD: 8 body functions, 35 activities and participation, and 29 environmental factors, and 111 ICF codes for the comprehensive Core Set for autism: 1 body structure, 20 body functions, 59 activities and participation, and 31 environmental factors. Age-specific and brief Core Sets were also published for each diagnosis, consisting of a certain set of codes from the comprehensive Core Sets. The Core Sets for autism and ADHD were recently revised. The revision led to 27 ICF added codes in the comprehensive ADHD Core Set and 12 ICF codes added and two removed in the comprehensive autism Core Set. The revised comprehensive Core Set for ADHD now contains 98 ICF codes and 121 ICF codes in the comprehensive Core Set for autism (Bölte et al., 2024a, 2024b).

## Method

A scoping review methodology was conducted in accordance with Arksey and O’Malley (Arksey & O’Malley, 2005) and an extension by Levac et al. (2010). The review was pre-registered (https://doi.org/10.17605/OSF.IO/8S6WG).

### Study selection

To be included in the review, articles were required to (1) include individuals diagnosed with autism and/or ADHD according to the *ICD* or *Diagnostic and Statistical Manual of Mental Disorders* (*DSM*) (American Psychiatric Association, 2013; WHO, 2022). Studies including other diagnoses were included if they included a subset of individuals with autism and/or ADHD where results could be separated; (2) discuss and/or apply the ICF, the ICF-CY, or ICF Core Sets for autism or ADHD; (3) be written in English or Swedish; and (4) be a journal article, report, commentary, editorial, or policy document. No limitations were placed on the publication date. Publications were excluded if they were theses or dissertations or made reference to the ICF but did not use it to inform analysis/results or discussion.

### Search strategy

A literature search was performed in the following databases: Medline, Embase, ERIC, Web of Science, PsycINFO, and CINAHL. A simplified version was also developed for Google Scholar. The last searches were conducted in August 2023. The search strategy was developed in Medline (Ovid) in collaboration with librarians at the Karolinska Institutet University Library. For each search concept Medical Subject Headings (MeSH-terms) and free text terms were identified. To validate the search strategy, 17 articles (positive list) were identified and checked for inclusion in the search results. The search was then translated into the other databases. In all databases, the following search term combinations were used in the subject terms (SU) field: (autism or autistic or “Autism Spectrum Disorder” or “Autism Spectrum Condition” or ASD or ASC or Asperger or “Asperger Syndrome*”) and (ADHD or “Attention Deficit Disorder” or “Attention Deficit Hyperactivity Disorder”) and (ICF or “International Classification of Functioning” or “International Classification of Functioning, Disability and Health” or “ICF-CY” or “Core Set”). No language restriction was applied, and databases were searched from inception. De-duplication was performed using the method described by Bramer et al. (2016). DOIs were also compared to identify remaining duplicates. The full search strategies for all databases are available in the Supplemental Material. Identified articles were imported into the reference management software Endnote 20 (The EndNote Team, 2013).

### Data extraction

Characteristics of included studies were extracted and summarized using a standardized form with the following headings: (1) Condition; (2) Age group; (3) ICF version; (4) Aim; (5) Application of the ICF; (6) Application context; (7) Country; and (8) Study design. Two authors (L.A. and M.B.) discussed one article together before performing extraction for the remaining sources. One author (L.A.) performed the extraction for all the sources, while another (M.B.) conducted extraction on a subset for reliability verification.

### Collating and summarizing results

Narrative synthesis (Popay et al., 2006) was used to summarize and analyze the findings from the included studies. Narrative synthesis allows for including different forms of evidence within a review (Popay et al., 2006). Data were thematically organized, and findings were analyzed individually and in relation to one another. Themes and sub-themes were then synthesized to provide a comprehensive overview of the existing evidence. The outline of themes was reviewed with the other members of the research team, who discussed and refined themes to achieve consensus.

### Methodology quality review

Standard quality assessment criteria for evaluating primary research papers from a variety of fields (QualSyst; Kmet et al., 2004) were used to assess the validity and reliability of the studies. Studies that did not describe primary research were excluded from the quality assessment. One author (L.A.) conducted quality assessments for the sources, and another (M.B.) conducted quality assessments on a subset for reliability verification. The QualSyst scores were interpreted as follows: 80% or higher as strong quality, 60%–79% as good quality, 50%–59% as adequate quality, and 50% or lower as poor quality. The quality of the studies ranged between poor (40%) and strong (100%). Studies were not excluded based on the quality assessment, but the findings were interpreted with consideration of the quality of the evidence. The quality of the studies is presented in Supplemental Table 1.

### Community involvement

Members of the autism community were not involved in the design, implementation, or interpretation of this study.

## Results

### Study selection

**Figure 2.**
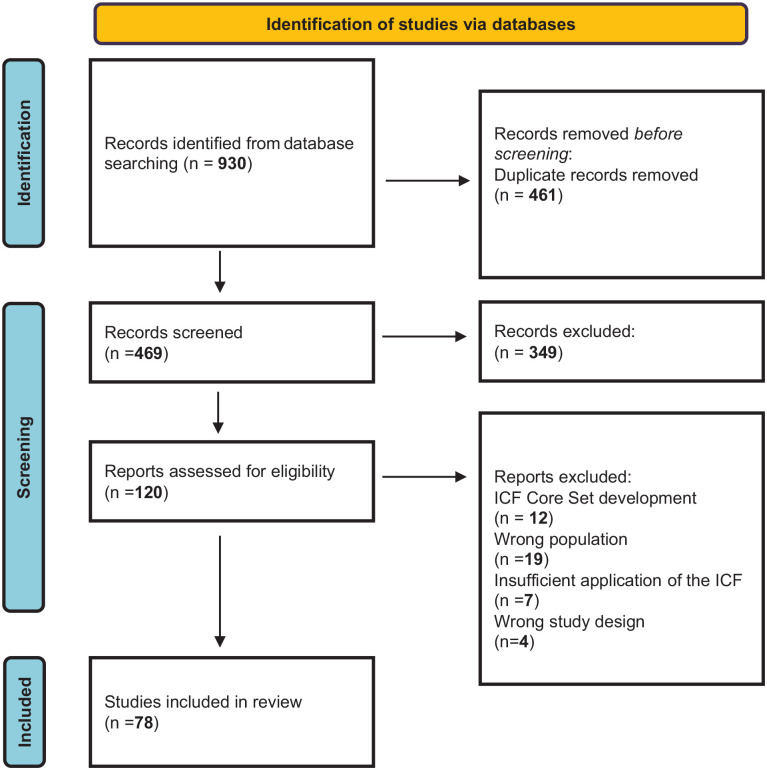
Flow diagram of the literature search.

The searches retrieved 930 documents, 469 following the removal of duplicates, which were screened at the title and abstract level (Figure 2). Two authors (L.A., M.B.) read the abstracts independently and removed those not fulfilling the inclusion criteria (*k* = 349). Agreement at the abstract level between the two reviewers was 85%, with a Cohen’s kappa of 0.68, which is interpreted as moderate agreement (McHugh, 2012). Disagreements were resolved by discussion. A total of 120 documents were subjected to full-text screening for eligibility, resulting in 30 being excluded (Figure 2). Twelve studies described the development of the autism and ADHD ICF Core Sets, summarized elsewhere, and are not included in the analysis. Supplemental Table 1 reports the characteristics of the 78 studies that were included in the final analysis.

### Study characteristics

**Figure 3.**
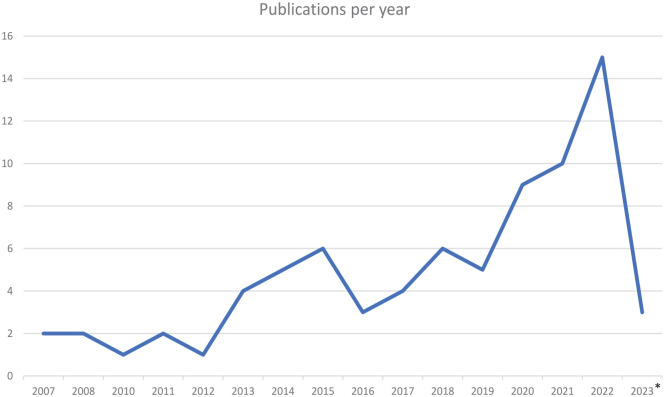
The number of included articles in this review, organized after a year of publishing. Note that 2023 is incomplete since the data search was conducted in August 2023.

The included studies are summarized in Supplemental Table 1. Most studies included populations of individuals with autism (*k* = 63), with less focus on ADHD (*k* = 8) or autism and ADHD (*k* = 7). In addition, studies primarily examined child or adolescent populations (*k* = 54). Studies were most frequently conducted in high-income countries (*k* = 64), with Australia (*k* = 14), Sweden (*k* = 11), and the United States (*k* = 11) being most represented. A range of study designs were employed, most commonly reviews, qualitative and quantitative study designs, and discussion papers. Over the years, there was an increase in the number of publications (see Figure 3).

### ICF version

The majority of the articles in this review reported using or made reference to the ICF (*k* = 35) or the ICF-CY (*k* = 21). Several articles used the ICF Core Sets (*k* = 14). Of these, most articles used the ICF Core Sets for autism (*k* = 11), and a few used a combination of the Core Sets for autism and ADHD (*k* = 3). No article used only Core Sets for ADHD. A few articles used a combination of multiple versions (*k* = 8) (Supplemental Table 1). For articles using or referencing the Core Sets, some used the Comprehensive version (*k* = 6), others used the Brief version (*k* = 5), while a few used a combination of both versions (*k* = 3). Others did not provide information regarding the version used (*k* = 6).

### Application methods

The ICF, ICF-CY, and Core Sets were used in a variety of ways by the included studies, ranging from theoretical or conceptual use to more concrete methodological application. A majority of studies used the ICF as a conceptual or guiding framework for various purposes, including to guide data collection for example interview questions, to guide analysis, and to describe outcomes. A substantial number of the studies also performed an “ICF linking” process. ICF linking normally follows standardized guidelines for translating information into ICF codes (Cieza et al., 2019). This process was used in some qualitative studies, whereby responses from participants were translated into ICF terminology. Other studies also performed linking to improve the comparability of findings across studies in the context of review articles. Several articles also linked existing measures to the ICF to examine the content validity of measures. Other studies, though not performing the specific linking process outlined by the ICF research branch, broadly mapped findings to ICF domains to provide an overview of outcomes. Finally, in several studies, the ICF was used to develop measures of functioning.

### Application contexts

**Figure 4.**
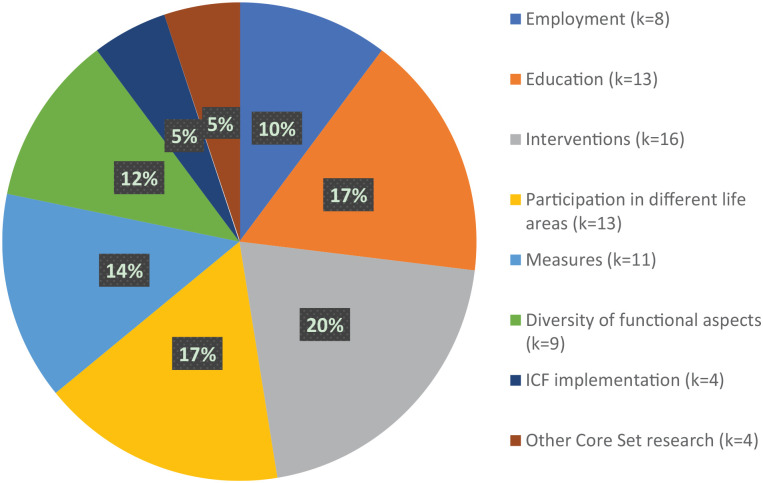
Application contexts of included studies (*k* = 78).

The ICF and its various versions were applied in several contexts (Figure 4).

### Education

Thirteen studies applied the ICF in education contexts with a spread across education levels, from preschool children to university students. A common theme arising from studies applying the ICF in the education context was the influence of environmental factors on academic outcomes and adjustment for autistic and ADHD youth, but also the lack of consideration thereof (Adolfsson & Simmeborn Fleischer, 2015; Aljunied & Frederickson, 2014; Castro et al., 2014; Gaona et al., 2020). Aljunied and Frederickson (2014) identified that even when autistic children required environmental support, those provided were often inadequate. Two literature reviews investigated difficulties experienced by autistic and ADHD students in the context of higher education (Emmers et al., 2017; Jansen et al., 2018). Emmers et al. (2017) summarized their results according to ICF domains and found that body functions and body structures domains were well covered, but information about other domains (environment and activity and participation) was scarcer. Jansen et al. (2018) conducted a literature review and focus groups to explore challenges facing higher education students using the ICF as a guiding framework. They found that no study had examined environmental or personal factors.

Several studies examined how the ICF could be used to develop and plan support and accommodations for youth with autism and ADHD. In their review, Leifler et al. (2021) explored accommodations in the learning environment, finding a lack of consideration of environmental influences to support autistic youth engagement. Similarly, educational goals for autistic youth were largely found to exclude environmental factors (Rama et al., 2019). The ICF was also used in one study to develop strategies that can facilitate communication between students and university coordinators regarding accommodation needs (Adolfsson & Simmeborn Fleischer, 2015).

One environmental support often mentioned was peer specialist mentoring among autistic university students (Thompson et al., 2020, 2021). The ICF was used as a guiding framework to coherently study the active ingredients of these mentoring programs, with the researchers arguing that an important benefit of using the structure of the ICF is the holistic approach as a help to both identify the challenges and the strengths of autistic individuals as well as contextual factors influencing the program.

One article discussed the ICF as a conceptual framework for describing functional problems in education associated with ADHD (Loe & Feldman, 2007). The researchers concluded that the ICF is suitable to evaluate treatments in terms of whether they improve the ICF domain’s body functions, including attention and executive functions; activities, including increasing learning and applying knowledge; and participation, including moving across educational levels or from school for work. Finally, one study investigated the determinants of behaviors, school adjustment, and QoL in autistic children (Schneider et al., 2022). School adjustment was measured using an ICF-based measure. Findings showed that higher autistic-like traits were associated with a better course of school adjustment whereas co-occurring psychiatric conditions were associated with worse outcomes in adjustment in autistic children.

### Employment

Eight studies addressed employment. All articles focused on autistic individuals, except one which addressed employment for people with ADHD. This sole study found that work performance in individuals with ADHD was predicted partly by body functions such as sensory sensitivity and sleep quality. Their findings underline the importance of a comprehensive analysis, including not only diagnostic symptoms but also body functions to understand work performance in individuals with ADHD (Grinblat & Rosenblum, 2022).

Four articles used the ICF to identify factors that facilitate and hinder employment for autistic individuals (Black et al., 2019; Dreaver et al., 2020; Khalifa et al., 2020; Scott et al., 2015). A majority of the studies emphasized the importance of examining the environment’s impact when understanding and improving employment for autistic individuals (Black et al., 2019; Dreaver et al., 2020; Khalifa et al., 2020; Scott et al., 2015). Both the ICF and the ICF Core Sets for autism were concluded to be useful in holistically examining employment in autism and ADHD (Black et al., 2019; Dreaver et al., 2020; Grinblat & Rosenblum, 2022; Khalifa et al., 2020; Scott et al., 2015, 2019).

Literature relating to employment for autistic adults was systematically reviewed in two articles. One used the ICF as a theoretical framework to guide the examination of workplace accommodations (Khalifa et al., 2020), while the other extracted and linked meaningful concepts relating to employment interventions to the ICF Core Sets for autism (Scott et al., 2019). Both articles highlighted the lack of intervention studies considering environmental factors but instead focused on changing the behavior of autistic individuals. For example, of the 36 intervention-based studies found in the study by Scott et al. (2019), not one addressed environmental factors as the main target of the intervention. However, the reviews show that environmental factors play a pivotal role as barriers and facilitators and therefore there is a critical need for interventions that target contextual factors if employment outcomes are to be improved.

Two studies used the ICF when investigating the efficacy of a strengths-based program aimed at providing pathways to future employment opportunities (Lee et al., 2020, 2024). In one study, the outcomes of the program were evaluated using a questionnaire developed by the researchers based on the categories of the ICF Core Sets for autism (Lee et al., 2020), while the other used the ICF as a guiding framework for qualitative data analysis (Lee et al., 2024). They reported that the program led to parent-reported improvements in ICF chapters related to community, interpersonal interactions and relationships, and major life areas (Lee et al., 2020), and identified a range of body functions and structures, activities and participation, and contextual factors that influenced their participation in the program (Lee et al., 2024).

### Interventions

The ICF has been applied in various ways to inform the development and evaluation of interventions, services, supports, and goal-planning (Supplemental Table 1).

Five studies were review articles that used the ICF to guide their synthesis (*k* = 3) or linked findings to the ICF (*k* = 2). Peters and Wood (2017) categorized outcomes observed from engagement in equine-assisted therapies. They found that 37% of outcomes were categorized as activity and participation and 30% as body functions. Activities and participation, as opposed to body functions, were discussed as relating more closely to the everyday life needs of autistic individuals, with the authors using their mapping to discuss implications for outcome selection that aligns with community needs. A second review, also exploring equine-assisted therapy by conducting more explicit linking to the ICF, found that most evidence for equine-assisted therapy was related to body function or body structure outcomes (Helmer et al., 2021). Other reviews used the ICF taxonomy to conceptualize executive function-related difficulties in autism, identify potential assistive technologies (Desideri et al., 2020), and synthesize studies describing the underlying mechanisms of handwriting in autistic children (van den Bos & Rosenblum, 2023). The final review article linked studies to the ICF using established linking rules to examine the measurements and outcomes associated with wearable technologies to support functioning in autism (Black et al., 2020).

The ICF was used in the context of intervention goals by five studies. Most studies (*k* = 4) used the ICF to inform goal-setting or therapeutic objectives. For example, using the ICF categories to develop goal cards (Angeli et al., 2021), or to formulate therapeutic goals in the context of robot-assisted interventions (Huijnen et al., 2016; Robins & Dautenhahn, 2014; Robins et al., 2012). A single study used the ICF as a means to examine the focus of occupational therapy intervention goals for autistic children. By coding the content of occupational therapy goals to the ICF, the authors identified that the goals mainly focused on activities and participation (Schaaf et al., 2015).

Three articles discussed how the ICF may be used to guide practice in various contexts (Adams, 2022; Gray et al., 2008; Ren & Hua, 2022). Adams (2022) discussed the assessment and management of feeding difficulties in autistic children using the ICF and suggested that an effective intervention for feeding difficulties entails a holistic perspective that considers challenges in a biopsychosocial context. Ren and Hua (2022) discussed the application of the ICF in speech and sport rehabilitation and environmental interventions, highlighting that the ICF enables a more comprehensive assessment and recognition of the diverse range of difficulties faced by autistic children. Finally, Gray et al. (2008) discussed communication about autism for healthcare professionals. According to the authors, primary care personnel can effectively utilize the ICF framework to communicate about autism to clients and families in a sensitive and respectful manner.

Other applications of the ICF in relation to intervention were also seen. One study described a digital platform designed to enhance communication about autism between autism families, schools, and healthcare (Vallefuoco et al., 2021). Here, the ICF was used as a framework to describe the individual’s functioning with a shared language to everyone involved. Vallefuoco et al. (2022) utilized an ICF-inspired form based on the activity/participation domain to assess the impact of virtual training with serious games on supermarket shopping. Post-training, all participants demonstrated improved scores in the monitored ICF-CY, particularly in areas such as attention and orientation abilities. In a case study, Mohan et al. (2019) used the ICF to examine the biopsychosocial influences contributing to challenges experienced by an autistic child. This framework was subsequently used to guide intervention considerations when aiming to facilitate language development and communication through technological assistance.

### Measures

The ICF was used to validate or develop measurements of functioning in 11 articles. All articles but one concerned children or youth. In some articles, the ICF was used to investigate the content validity of measures. Three articles linked measures to the ICF according to the ICF research branch and WHO guidelines. Two investigated the content validity of functioning measures, and one examined measures of autistic traits. In the two studies examining measures of functioning, commonly used measures of functioning in autism (Hayden-Evans et al., 2022) and NDCs (D’Arcy et al., 2022) were compared to the ICF and the Core Sets for autism, ADHD, cerebral palsy, early delay and disabilities, and a combined “early neurodevelopmental” set generated by the authors. These linking exercises revealed that the measures covered a limited range of functioning when compared to the Core Sets.

Castro et al. (2013) linked several autism screening and diagnostic measures to the ICF to determine the functional information captured by these measures. They found that the measurements provided information on various functional aspects such as the ICF domain’s body functions, and activities and participation that were not typically captured when scoring the measures. They suggest that through using the ICF, traditionally “symptomology” based measures can capture aspects of functioning that may be important to clinical practice.

Another article also examined classification measures used in NDCs, including autism. Although not linking explicitly to the ICF, they broadly mapped the ICF constructs (e.g., performance, capacity, and body functions) captured by commonly used measures. Similar to the findings of Castro, they found that primarily body functions were captured, and that measures typically captured either performance or capacity, and rarely both (Jeevanantham & Bartlett, 2017). Using a similar mapping exercise, Munsell and Coster (2022) categorized measures to identify aspects of participation, using the ICF as a framework to provide a systematic description of outcomes and to examine whether all relevant areas of participation had been assessed. They found that even though the measures covered a breadth of participation, they often lacked detail and had inconsistent terminology. The authors noted that the ICF framework has the potential to provide a common language as well as a more comprehensive perspective on participation. The ICF has also been used to develop functional measures and questionnaires. For example, an ICF-based classification system that classifies social communication in autistic children has been developed (Di Rezze & Rosenbaum, 2016). However, this classification system has received some criticism (Vries & Bölte, 2016). Another ICF-based tool has been developed in order to underline the role of engagement and environment in determining functioning, the Matrix for Assessment of Activities and Participation (Castro & Pinto, 2015). Moreover, two ICF-based questionnaires for autistic children have also been developed (Gan et al., 2013; Napoli et al., 2021). Finally, Tahir used an operationalized version of the ICF Core Sets for autism to identify functioning profiles in autistic children in a tertiary care hospital, finding it helpful in providing information beyond diagnostic criteria and helping to assess the environment’s impact on the child.

### Participation in different life areas

Thirteen studies used the ICF to examine participation across different life areas in autistic and ADHD children, youth, and young adults aged 3 to 30 years. Many studies addressed autistic individuals’ experiences or difficulties regarding participation, in areas such as out-of-school activities (Askari et al., 2015), communication and community participation (Poon, 2011), social inclusion (Krieger et al., 2018), employment and postsecondary education (Liptak et al., 2011), and physical activity (Bishop et al., 2018; Bremer et al., 2020). Through using the ICF, studies were able to identify a range of both hindering and facilitating factors influencing this participation. Hindering factors for participation were reported to be, for example, repetitive or stereotyped behaviors (Askari et al., 2015; Germani et al., 2017), limited gross and fine motor ability (Askari et al., 2015), sensory sensitivity (Askari et al., 2015), and functional challenges (Bremer et al., 2020). Reported facilitating factors were for instance social and communicative ability (Gan et al., 2014; Germani et al., 2017; Liptak et al., 2011; Thompson et al., 2018), social support (Askari et al., 2015; Krieger et al., 2018; Liptak et al., 2011) and following routines (Coussens et al., 2020; Germani et al., 2017). The importance of environmental factors to understand and support participation in individuals with autism and ADHD was highlighted (Askari et al., 2015; Coussens et al., 2020; Krieger et al., 2018; Liptak et al., 2011; Thompson et al., 2018), with the studies suggesting that diagnosis and its clinical manifestations are not the only important factor influencing participation, emphasizing the influential role of the environment.

Some studies used the ICF to examine how autistic individuals experience aspects of participation. Clement et al. (2022) set out to deepen the understanding of how autistic youth, their families, and autistic mentors experience meaningful participation. They used the ICF as a guiding framework, concluding that the ICF participation domain assesses only a limited range of aspects that impact participation. Consequently, they suggested that, for instance, sensory aspects should be more clearly represented in the domain. Moreover, Fridell et al. (2022) explored the experiences and impact of COVID-19 on the autistic community by linking autistic adults’ experiences to the ICF. The results revealed that almost half of coded outcomes related to activities and participation. Hence, the ICF informed an understanding of the impact that COVID-19 had on the participation of autistic individuals. Finally, two studies incorporated ICF-based measures to assess participation in children diagnosed with autism and ADHD. Lin et al. (2022) conducted a cross-diagnostic study over 3 years, using an ICF-based measure to evaluate various aspects of participation in children with learning problems including autism and ADHD. Germani et al. (2017) examined social participation in autistic children by developing a tool using 18 (of 572 possible descriptors) from the activity and participation domain of the ICF-CY. They found that ICF constructs most important for social participation were regulating behaviors within interactions, responding to demands, and following routines. Finally, one study utilized an ICF-based questionnaire to construct structural equation models examining factors associated with participation in autistic children (Gan et al., 2014).

### Diversity of functional aspects

The ICF was used in studies describing the diversity of functional aspects of autism and ADHD in nine articles. These studies focused on a broad range of areas, such as gender differences (Lundin et al., 2021), the impact of contextual factors on functional ability (Viljoen et al., 2019), parental perspectives on functioning (Viljoen et al., 2021), the relationship between impairments, functional skills and the management of life tasks (Kao et al., 2015), the relationship between ADHD symptoms and functional impairment (Garner et al., 2013; Riley et al., 2008), support needs of autistic children (Evans et al., 2022), sensory characteristics in autistic children (Mikami et al., 2018), and factors associated with QoL in autism (Jacoby et al., 2022).

The findings of several studies were linked to the ICF using rules outlined by the ICF research branch (*k* = 3). For example, Lundin et al. (2021) explored professional perceptions of functional gender differences in autism. They found several unique aspects of functioning in autistic females, but through linking to the ICF, they found that the vast majority (97%) of factors identified as relevant for gender differences in autism were covered by the comprehensive Core Sets, concluding that the Core Sets were relevant to examining gender differences in autism. The two other studies linking findings to the ICF explored parents’ perspectives on functioning in autism. One used the ICF in a cross-cultural comparison of functioning in South Africa and Sweden (Viljoen et al., 2019). Despite the contrasting nature of the two countries, they found that factors relevant to functioning in autism were largely similar across cultures. Discrepancies were more frequently observed in relation to body functions and activities and participation, as opposed to environmental factors. The other study investigated parents’ perspectives on functioning in high-income versus low-/middle-income countries in a global scoping review (Viljoen et al., 2021). Through linking studies conducted in low- and middle-income countries to the ICF, it was revealed that high-income countries encompassed all ICF domains while functional themes from low- or middle-income countries mainly focused on environmental and personal factors.

Two studies utilized the ICF as a guiding framework when examining the relationship between functioning and other outcomes. One study examined the relationship between impairments, functional skills, and the management of life tasks in autistic adolescents, using the ICF framework to select model variables whereby existing measures, such as the Social Communication Questionnaire (SCQ) (Rutter, 2013) and The Pediatric Evaluation of Disability Inventory-Computer Adaptive Test–Autism Spectrum Disorders (PEDI-CAT-ASD) (Kramer et al., 2012), were broadly categorized as capturing specific body functions or activities and participation (Kao et al., 2015). They found that the management of life tasks was more strongly associated with functional skills of daily activities than IQ or the severity of autism symptoms. In other studies, the relationship between ADHD symptoms and functional impairment in children with ADHD was evaluated (Garner et al., 2013; Riley et al., 2008). Garner et al. (2013) found that symptoms of inattention predicted impairments in learning and applying knowledge when parents rated their child’s impairments in ICF areas relating to applying knowledge. Riley et al. (2008) found no differences in functional impairment when comparing children with different types of ADHD (hyperactive-impulsive type and combined type).

Jacoby et al. (2022) examined factors influencing QoL in children with a range of conditions, including autism. In a regression tree analysis that used the ICF domains as independent variables, they found that sleep and community participation were important factors associated with QoL. The ICF was also used as an overarching framework to explore the support needs of autistic individuals and analyze results based on ICF domains (Evans et al., 2022). The support requirements encompassed various domains of the ICF, including body functions, activities and participation, and environmental factors. The authors conclude that this highlights the intricate nature of the experienced challenges. Finally, Mikami et al. (2018) examined sensory characteristics in autistic children and coded the results to ICF domains. The authors found that body functions, activities and participation, and environmental factors were represented without large deviations.

### ICF implementation

Four articles discussed the benefits of using the ICF in autism and ADHD research and practice (Bölte, 2023; Bölte, Lawson, et al., 2021; Kintzinger, 2021; Üstün, 2007). Arguments for the utility of the ICF included its holistic understanding of autism, representing a shift from a biomedical model approach to a biopsychosocial one (Bölte, 2023), its potential to reconcile the biomedical and the neurodiversity paradigms (Bölte, Lawson, et al., 2021) and its potential to be used to understand the nature of ADHD further (Üstün, 2007). Finally, an autistic self-advocate (Kintzinger, 2021) argued that there are many advantages for autistic people to use the ICF, as it is a more universal model of functioning than the medical or social model of disability. Therefore, the author argues that the ICF is well-suited not only to be a classification tool but also to be used in policy and research.

### Other Core Set research

Two articles regarding research on the official ICF Core Sets for autism and ADHD were found. Schiariti et al. (2018) compared the ICF Core Sets for autism, ADHD, and cerebral palsy, concluding that even though commonalities exist between the Sets, they do indeed capture unique functional aspects, providing justification for condition-specific Core Sets. Furthermore, a systematic review verified the number of Core Sets of ICF-CY that had been developed, which included Sets for autism and ADHD (Tofani et al., 2023).

Two articles described the process of developing other and preliminary Core Sets for autistic children (0 to 2 and 3 to 6 years) (Castro & Pinto, 2013) and Swedish Core Set for ADHD adults (Söderström et al., 2014). Castro and Pinto (2013) identified core functioning features in autistic children using a Delphi technique where they collected the opinions of experts in child development and autism through a three-round online survey. The final version of the Core Set for children 0 to 2 years contained 14 ICF categories and for children 2 to 6 years, 65 ICF categories. The development of the Swedish Core Set for adults by Söderström et al. (2014) contained two phases: a Delphi exercise with participants from various professions and a patient organization, and a consensus conference with participants with experience in ADHD and the ICF. This process led to the development of a national comprehensive Core Set for adults with ADHD containing 66 categories.

## Discussion

This review demonstrates the broad and varied ways in which the ICF has been used to facilitate biopsychosocial investigation in autism and ADHD, capable of capturing more comprehensive perspectives that may more readily align with the everyday lives of individuals with NDCs and reconciling biomedical and neurodiversity approaches.

The use of the ICF in research has increased since its publication in 2001, perhaps reflecting the ongoing paradigm shift from traditional one-sided medical approaches, toward more comprehensive perspectives within the field. This shift is arguably more evident in relation to autism where the neurodiversity movement originated, and indeed most research identified in this review was in relation to autism, potentially reflecting these broader community trends. Despite this shifting paradigm, clinical practice and research are still largely guided by the internationally recognized standards of the *DSM* and the *ICD*, whereby the individual’s diagnosis remains the primary focus for discussing NDCs. This dominance may, in part, be explained by the earlier establishment of these classifications compared to the more contemporary ICF. While the ICF is not the sole model for capturing a biopsychosocial perspective of the individual, it remains the only globally recognized and comprehensive model and framework that is specifically designed to complement other classifications. Despite the increasing uptake of the ICF, we identified 78 articles, which may be less than expected given that the ICF was published over two decades ago. Alongside the ongoing dominance of the biomedical model, the comprehensive nature of the ICF may have also detracted from its use. The ICF Core Sets may assist in seeing the ICF more readily adopted in research and practice.

The biopsychosocial and “diagnostically agnostic” approach of the ICF appears to be advantageous in facilitating research that embraces neurodiversity because it is not concerned with diagnostic status, encompasses factors outside of symptoms/traits, and looks more globally at the individual, including their challenges and strengths, as well as environmental factors, operationalizing functioning as the interplay between an individual and their context (Dwyer, 2022). However, it is critical to note that it is necessary to conform to the underlying principles of the ICF, and not simply refer to or use the ICF, for research to be neurodiversity-affirmative. We identified several studies that, while using the ICF, did not appear to conform to its underlying principles. These principles emphasize that functioning should be universally applicable, regardless of one’s health condition, and should not be concerned with symptoms, but rather how someone functions as a result of an interaction with their environment, and respecting autonomy and participation. Some of these studies organized their outcomes based on ICF domains, but the collected data primarily focused on co-occurring diagnoses, symptom severity, or disabilities. Furthermore, the ICF philosophy also emphasizes the examination of strengths, but it seems to be seldom employed for that purpose. It appears that the impact of the medical perspective still influences research methodologies and interpretation, even when the explicit aim is to utilize the ICF framework. Thus, researchers and clinicians must remain cognizant of the ICF’s underlying principles in applying it in research and practice and should avoid merely using the ICF as a superficial indicator of conducting neurodiversity-affirmative research when it may not align with the true intentions.

Our findings show that the ICF has been utilized to develop several measures. While this is explicable due to the extensiveness of the ICF which makes it impractical to use in many contexts, there is a potential risk associated with developing numerous ICF-based measures. First, one objective of the ICF is to establish a biopsychosocial model with a shared language to enable communication across professions and contexts. However, unless these measures are developed with explicit reference to the conceptual framework of the ICF and its underlying philosophies, this shared language may become diluted. Second, many measures developed based on the ICF selected particular ICF components to measure, with little transparency of decisions underlying this selection process, making it unclear if these measures truly capture relevant aspects of functioning. Finally, the psychometric underpinnings of these measures are unknown. Other measures commonly used to assess functioning such as the Adaptive Behavior Assessment System (Harrison & Oakland, 2000) and the Vineland Adaptive Behavior Scales (Sparrow & Cicchetti, 1989) are more widely utilized and have a stronger psychometric basis, however, it is important to note that these assessment scales only cover a restricted scope of the ICF framework, predominantly concentrating on body functions and the activity and participation domain (D’Arcy, 2022; Gleason & Coster, 2012). Utilization of the ICF Core Sets for autism and ADHD may overcome many of these issues associated with ICF-based measures. The ICF Core Sets for autism and ADHD were developed using a rigorous and methodologically sound process, with strong supporting evidence suggesting that they capture factors relevant to functioning in autism and ADHD globally. Thus, it would follow that the Core Sets may provide a logical basis for developing a standardized and globally useful measure that could be used in both research and practice. In lieu of using measures based on the ICF and its Core Sets, there are also possibilities to translate existing measures (which may be symptomology-based) to ICF nomenclature. Such a process may provide opportunities for existing measures to be used in more neurodiversity-affirmative ways while also enabling harmonization across measures (Black et al., 2023).

The ICF is designed to be applicable to all individuals, and the development of the autism and ADHD Core Sets explicitly sought to include categories of relevance across developmental stages (Bölte et al., 2024a, 2024b). However, despite the ICF and the Core Sets being suitable for all age groups, most articles included in this review focused on children and youth with NDCs. Childhood serves as a crucial period in an individual’s life, and addressing and supporting challenges and problems during this phase can yield substantial benefits for both the individual and society as a whole. This observation also reflects broader research and funding patterns where most focus has been placed on childhood, although there are now some shifts in this funding profile (Cervantes et al., 2021; Sibley et al., 2023). However, it is imperative for research on NDCs to encompass the entire lifespan to benefit individuals with autism and ADHD across all age groups. This review demonstrates that although the ICF has been effectively applied in numerous areas, there are still noteworthy topics, not least related to adults and the elderly, where the ICF can be highly valuable, for example, to explore transition periods such as becoming a parent or going into retirement.

Few studies examined personal factors, which may, in part, be explained by the fact that there currently exists no WHO-endorsed classification system for personal factors as part of the ICF. Jansen et al. (2018) was one of the few studies that explicitly explored personal factors in their literature review, finding that no study examining challenges facing higher education students explored personal factors. Personal factors likely play an important role in the functioning of individuals with autism and ADHD. For example, gender (designated a personal factor in the ICF) has been shown to influence the presentations and impacts of autism and ADHD in females/women (Faheem et al., 2022; Lundin et al., 2021). Moreover, individual attitudes, including empowerment and self-efficacy, influence engagement in and effectiveness of healthcare services (Park et al., 2018). Despite the ICF not explicitly categorizing these factors, it is nonetheless encouraged to consider them in healthcare assessments. Although not officially endorsed by the WHO, some personal factor classification systems have been developed to complement the ICF, such as those proposed by Grotkamp et al. (2020) and Geyh et al. (2019), that may prove useful for future research.

The interpretation of the results must be considered in light of several limitations. First, this study is restricted to articles published in English and Swedish, resulting in the exclusion of studies written in other languages. Consequently, it is possible that this review does not encompass all published materials relating to the application of the ICF in autism and ADHD. Furthermore, the interpretation of the findings in this review was likely influenced by the backgrounds of the research team.

## Conclusion

The adoption of the ICF and its Core Sets for autism and ADHD demonstrates its potential in promoting research that embraces neurodiversity, offering a comprehensive view that emphasizes the impact of the environment and participation in daily life and society. The studies analyzed in this review consistently identify the significant influence of environmental factors on functioning, even though these factors are seldom taken into consideration. The ICF possesses the capacity to capture the diverse range of biological, psychological, and social factors that influence functioning across various contexts, such as education and employment. This capability allows research to transcend the limitations of biomedical perspectives and aligns with neurodiversity-aligning paradigms. Future research endeavors should focus on facilitating the implementation and utilization of the Core Sets to enhance their effectiveness. Still, more progress is required before the ICF can exert as much influence as the *ICD* and the *DSM* in the understanding of NDCs.

## Supplemental Material

sj-docx-1-aut-10.1177_13623613241272044 – Supplemental material for Application of the international classification of functioning, disability, and health in autism and attention-deficit hyperactivity disorder: A scoping review Supplemental material, sj-docx-1-aut-10.1177_13623613241272044 for Application of the international classification of functioning, disability, and health in autism and attention-deficit hyperactivity disorder: A scoping review by Lovisa Alehagen, Sven Bölte and Melissa H Black in Autism

sj-xlsx-2-aut-10.1177_13623613241272044 – Supplemental material for Application of the international classification of functioning, disability, and health in autism and attention-deficit hyperactivity disorder: A scoping review Supplemental material, sj-xlsx-2-aut-10.1177_13623613241272044 for Application of the international classification of functioning, disability, and health in autism and attention-deficit hyperactivity disorder: A scoping review by Lovisa Alehagen, Sven Bölte and Melissa H Black in Autism
