# Supplementary material for: Application of the international classification of functioning, disability, and health in autism and attention-deficit hyperactivity disorder: A scoping review
Source: Autism. 2024 Aug 25;29(2):310–28. doi: 10.1177/13623613241272044 (PMC11816479; doi:10.1177/13623613241272044)
Supplement: sj-docx-1-aut-10.1177_13623613241272044 – Supplemental material for Application of the international classification of functioning, disability, and health in autism and attention-deficit hyperactivity disorder: A scoping review [file sj-docx-1-aut-10.1177_13623613241272044.docx]

Documentation of search strategies

University Library search consultation group

Date: August 2023

Topic/research question: Use of the International Classification of Functioning, Health and Disability (ICF) in Autism an ADHD

Name of researcher(s): Melissa Black & Lovisa Alehagen

Librarian(s): Emma-Lotta Säätelä

Databases:

1. Medline (Ovid)
2. Embase (embase.com)
3. ERIC (ProQuest)
4. Web of Science Core Collection (Clarivate)
5. Psycinfo (EBSCO)
6. Cinahl (EBSCO)
7. Google Scholar (Publish or perish)

Total number of hits:

- Before deduplication: 930
- After deduplication: 469

Text that can be used in the Methods-section:

Search strategy

A literature search was performed in the following databases: Medline, Embase, ERIC, Web of Science, Psycinfo and Cinahl. A simplified version was also developed to work in Google scholar. The last searches were conducted 2023-08-07.

The search strategy was developed in Medline (Ovid) in collaboration with librarians at the Karolinska Institutet University Library. For each search concept Medical Subject Headings (MeSH-terms) and free text terms were identified. The search was then translated into the other databases.

No language restriction was applied and databases were searched from inception.

De-duplication was done using the method described by Bramer et al (1). One final, extra step was added to compare DOIs.

The full search strategies for all databases are available in the appendix.

References

1. Bramer, W. M., Giustini, D., de Jonge, G. B., Holland, L., & Bekhuis, T. (2016). De-duplication of database search results for systematic reviews in EndNote. *Journal of the Medical Library Association: JMLA*, 104(3), 240-243. doi: 10.3163/1536-5050.104.3.014


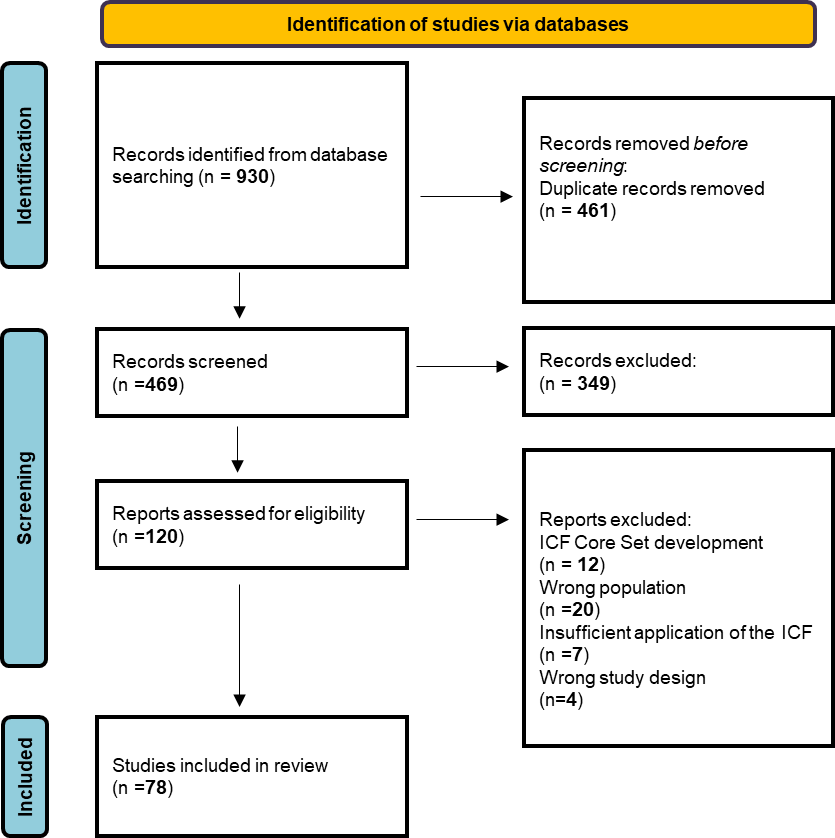


From: Page MJ, McKenzie JE, Bossuyt PM, Boutron I, Hoffmann TC, Mulrow CD, et al. The PRISMA 2020 statement: an updated guideline for reporting systematic reviews. BMJ 2021;372:n71. doi: 10.1136/bmj.n71. For more information, visit: http://www.prisma-statement.org/

1. Medline

| Interface: Ovid MEDLINE(R) ALL  Date of Search: 7 August 2023  Number of hits: 121  Comment: In Ovid, two or more words are automatically searched as phrases; i.e. no quotation marks are needed  The Ovid MEDLINE®️ database contains records with the following possible status besides MEDLINE: Publisher, In-Data-Review, In-Process and PubMed-not-MEDLINE records from NLM. | Field labels   - exp/ = exploded MeSH term - / = non exploded MeSH term - .ti,ab,kf. = title, abstract and author keywords - adjx = within x words, regardless of order - * = truncation of word for alternate endings |
| --- | --- |
| Database(s): **Ovid MEDLINE(R) ALL**1946 to August 04, 2023 Search Strategy:   \| **#** \| **Searches** \| **Results** \| \| --- \| --- \| --- \| \| 1 \| exp Autism spectrum disorder/ \| 42463 \| \| 2 \| exp Attention Deficit Disorder with Hyperactivity/ \| 34756 \| \| 3 \| (autism or autistic or asd or asc or asperger*).ti,ab,kf. \| 88331 \| \| 4 \| (adhd or attention deficit).ti,ab,kf. \| 42981 \| \| 5 \| or/1-4 \| 135413 \| \| 6 \| exp "International Classification of Functioning, Disability and Health"/ \| 855 \| \| 7 \| (icf* or international classification of functioning or "international classification of functioning, disability and health" or icf-cy or core set*).ti,ab,kf. \| 10268 \| \| 8 \| or/6-7 \| 10371 \| \| 9 \| 5 and 8 \| 121 \| | |

2. Embase

| Interface: embase.com  Date of Search: 7 August 2023  Number of hits: 230  Comment: Emtree is the controlled vocabulary in Embase | Field labels   - /exp = exploded Emtree term - /de = non exploded Emtree term - ti,ab,kw = title, abstract and author keywords - NEAR/x = within x words, regardless of order - * = truncation of word for alternate endings |
| --- | --- |
| \| No. \| Query \| Results \| \| --- \| --- \| --- \| \| #8 \| #4 AND #7 \| 230 \| \| #7 \| #5 OR #6 \| 14764 \| \| #6 \| icf*:ti,ab,kw OR 'international classification of functioning':ti,ab,kw OR 'international classification of functioning, disability and health':ti,ab,kw OR 'icf-cy':ti,ab,kw OR 'core set*':ti,ab,kw \| 14219 \| \| #5 \| 'international classification of functioning, disability and health'/exp \| 3511 \| \| #4 \| #1 OR #2 OR #3 \| 213007 \| \| #3 \| adhd:ti,ab,kw OR 'attention deficit':ti,ab,kw \| 60543 \| \| #2 \| autism:ti,ab,kw OR autistic:ti,ab,kw OR asd:ti,ab,kw OR asc:ti,ab,kw OR asperger*:ti,ab,kw \| 120677 \| \| #1 \| 'autism'/exp OR 'attention deficit hyperactivity disorder'/exp \| 164117 \| | |

3. ERIC

| Interface: ProQuest  Date of Search: 7 August 2023  Number of hits: 20 | Field labels   - MAINSUBJECT.EXACT = subject heading - MAINSUBJECT.EXACT.EXPLODE = exploded subject heading - Noft = anywhere except full text |
| --- | --- |
| \| [Set](https://www.proquest.com/recentsearches.recentsearchtabview.recentsearchesgridview:toggellistorder?site=eric&t:ac=RecentSearches) \| Search \| Results \| \| --- \| --- \| --- \| \| **S7** \| [[S5] AND [S6]](https://www.proquest.com/recentsearches.recentsearchtabview.recentsearchesgridview.scrolledrecentsearchlist.checkdbssearchlink:rerunsearch/17DAAEE4F00444B0PQ/None?site=eric&t:ac=RecentSearches) \| [**20**](https://www.proquest.com/recentsearches.recentsearchtabview.recentsearchesgridview.scrolledrecentsearchlist.checkdbssearchlink_0:rerunsearch/17DAAEE4F00444B0PQ/None?site=eric&t:ac=RecentSearches) \| \| **S6** \| [noft(icf* or "international classification of functioning" or "international classification of functioning, disability and health" or "icf-cy" or "core set*")](https://www.proquest.com/recentsearches.recentsearchtabview.recentsearchesgridview.scrolledrecentsearchlist.checkdbssearchlink:rerunsearch/1078F0D7FFE84167PQ/None?site=eric&t:ac=RecentSearches) \| [**490**](https://www.proquest.com/recentsearches.recentsearchtabview.recentsearchesgridview.scrolledrecentsearchlist.checkdbssearchlink_0:rerunsearch/1078F0D7FFE84167PQ/None?site=eric&t:ac=RecentSearches) \| \| **S5** \| [[S1] OR [S2] OR [S3] OR [S4]](https://www.proquest.com/recentsearches.recentsearchtabview.recentsearchesgridview.scrolledrecentsearchlist.checkdbssearchlink:rerunsearch/532BAF51741343D3PQ/None?site=eric&t:ac=RecentSearches) \| [**25,745**](https://www.proquest.com/recentsearches.recentsearchtabview.recentsearchesgridview.scrolledrecentsearchlist.checkdbssearchlink_0:rerunsearch/532BAF51741343D3PQ/None?site=eric&t:ac=RecentSearches) \| \| **S4** \| [noft(adhd or "attention deficit")](https://www.proquest.com/recentsearches.recentsearchtabview.recentsearchesgridview.scrolledrecentsearchlist.checkdbssearchlink:rerunsearch/CE28DBEBD8C24331PQ/None?site=eric&t:ac=RecentSearches) \| [**6,416**](https://www.proquest.com/recentsearches.recentsearchtabview.recentsearchesgridview.scrolledrecentsearchlist.checkdbssearchlink_0:rerunsearch/CE28DBEBD8C24331PQ/None?site=eric&t:ac=RecentSearches) \| \| **S3** \| [noft(autism or autistic or asd or asc or asperger*)](https://www.proquest.com/recentsearches.recentsearchtabview.recentsearchesgridview.scrolledrecentsearchlist.checkdbssearchlink:rerunsearch/9E1835D26432437DPQ/None?site=eric&t:ac=RecentSearches) \| [**20,212**](https://www.proquest.com/recentsearches.recentsearchtabview.recentsearchesgridview.scrolledrecentsearchlist.checkdbssearchlink_0:rerunsearch/9E1835D26432437DPQ/None?site=eric&t:ac=RecentSearches) \| \| **S2** \| [MAINSUBJECT.EXACT.EXPLODE("Attention Deficit Disorders")](https://www.proquest.com/recentsearches.recentsearchtabview.recentsearchesgridview.scrolledrecentsearchlist.checkdbssearchlink:rerunsearch/697DB6FBFCD14C9FPQ/None?site=eric&t:ac=RecentSearches) \| [**5,920**](https://www.proquest.com/recentsearches.recentsearchtabview.recentsearchesgridview.scrolledrecentsearchlist.checkdbssearchlink_0:rerunsearch/697DB6FBFCD14C9FPQ/None?site=eric&t:ac=RecentSearches) \| \| **S1** \| [MAINSUBJECT.EXACT("Autism Spectrum Disorders")](https://www.proquest.com/recentsearches.recentsearchtabview.recentsearchesgridview.scrolledrecentsearchlist.checkdbssearchlink:rerunsearch/3678C0D21BF14D2EPQ/None?site=eric&t:ac=RecentSearches) \| [**723**](https://www.proquest.com/recentsearches.recentsearchtabview.recentsearchesgridview.scrolledrecentsearchlist.checkdbssearchlink_0:rerunsearch/3678C0D21BF14D2EPQ/None?site=eric&t:ac=RecentSearches) \| | |

4. Web of Science Core Collection

| Interface: Clarivate Analytics  Editions = A&HCI , ESCI , SCI-EXPANDED , SSCI  Date of Search: 7 August 2023  Number of hits: 176 | Field labels   - TS/Topic = title, abstract, author keywords and Keywords Plus - NEAR/x = within x words, regardless of order - * = truncation of word for alternate endings   Note: the *Exact search*-function was used for all the searches |
| --- | --- |
| \| # \| Search Query \| Results \| \| --- \| --- \| --- \| \| 1 \| TS=(autism or autistic or asd or asc or asperger*) \| 126015 \| \| 2 \| TS=(adhd or "attention deficit") \| 60871 \| \| 3 \| #2 OR #1 \| 179628 \| \| 4 \| TS=(icf* or "international classification of functioning" or "international classification of functioning, disability and health" or "icf-cy" or "core set*") \| 15973 \| \| 5 \| #4 AND #3 \| 176 \| | |

5. Psycinfo

| Interface: EBSCO  Date of Search: 7 August 2023  Number of hits: 97 | Field labels   - DE = subject heading - TI = title - AB = abstract - KW = author keywords - Nx = within x words, regardless of order - * = truncation of word for alternate endings |
| --- | --- |
| \| **#** \| **Query** \| **Results** \| \| --- \| --- \| --- \| \| S7 \| S5 AND S6 \| 97 \| \| S6 \| TI ( icf* or "international classification of functioning" or "international classification of functioning, disability and health" or "icf-cy" or "core set*" ) OR AB ( icf* or "international classification of functioning" or "international classification of functioning, disability and health" or "icf-cy" or "core set*" ) OR KW ( icf* or "international classification of functioning" or "international classification of functioning, disability and health" or "icf-cy" or "core set*" ) \| 3,206 \| \| S5 \| S1 OR S2 OR S3 OR S4 \| 108,779 \| \| S4 \| TI ( adhd or "attention deficit" ) OR AB ( adhd or "attention deficit" ) OR KW ( adhd or "attention deficit" ) \| 40,163 \| \| S3 \| TI ( autism or autistic or asd or asc or asperger* ) OR AB ( autism or autistic or asd or asc or asperger* ) OR KW ( autism or autistic or asd or asc or asperger* ) \| 68,204 \| \| S2 \| DE "Attention Deficit Disorder" OR DE "Attention Deficit Disorder with Hyperactivity" \| 35,885 \| \| S1 \| DE "Autism Spectrum Disorders" OR DE "Autistic Traits" \| 55,223 \| | |

6. Cinahl

| Interface: Ebsco  Date of Search: 7 August 2023  Number of hits: 86 | Field labels   - MH+ = exploded Cinahl Heading - MH = non exploded Cinahl Heading - TI = title - AB = abstract - Nx = within x words, regardless of order - * = truncation of word for alternate endings |
| --- | --- |
| \| **#** \| **Query** \| **Results** \| \| --- \| --- \| --- \| \| S9 \| S5 AND S8 \| 86 \| \| S8 \| S6 OR S7 \| 5,967 \| \| S7 \| TI ( icf* or "international classification of functioning" or "international classification of functioning, disability and health" or "icf-cy" or "core set*" ) OR AB ( icf* or "international classification of functioning" or "international classification of functioning, disability and health" or "icf-cy" or "core set*" ) \| 4,944 \| \| S6 \| (MH "International Classification of Functioning, Disability, and Health") \| 2,901 \| \| S5 \| S1 OR S2 OR S3 OR S4 \| 63,796 \| \| S4 \| TI ( adhd or "attention deficit" ) OR AB ( adhd or "attention deficit" ) \| 18,381 \| \| S3 \| TI ( autism or autistic or asd or asc or asperger* ) OR AB ( autism or autistic or asd or asc or asperger* ) \| 37,515 \| \| S2 \| (MH "Attention Deficit Hyperactivity Disorder") \| 18,977 \| \| S1 \| (MH "Asperger Syndrome") OR (MH "Autistic Disorder") \| 29,740 \| | |

7. Google Scholar

| Interface: Publish or perish  Date of Search: 7 August 2023  Number of hits: First 200 | Field labels   - \| = OR - space = AND |
| --- | --- |
| **Keywords:**  adhd\|autism\|autistic\|"attention deficit"\|asperger "international classification of functioning"\|ICF | |
